# Health Resources and Strategies among Employed Women in Norway during Pregnancy and Early Motherhood

**DOI:** 10.1155/2015/705892

**Published:** 2015-04-05

**Authors:** Marit Alstveit, Elisabeth Severinsson, Bjørg Karlsen

**Affiliations:** ^1^Department of Health Studies, Faculty of Social Sciences, University of Stavanger, 4036 Stavanger, Norway; ^2^Centre for Women's, Family and Child Health, Faculty of Health Sciences, Buskerud & Vestfold University College, P.O. Box 235, 3603 Kongsberg, Norway

## Abstract

The number of women in paid employment is increasing. However, when becoming a mother for the first time, many seem unprepared for the challenge of balancing motherhood and work as well as for the impact on their health. The aim of this study was to investigate the health resources and strategies of employed women in Norway during pregnancy and early motherhood by means of salutogenic theory. A hypothetical-deductive interpretive approach based on Antonovsky's salutogenic theory was applied in a secondary analysis. A total of six themes were identified; three were classified as health resources when experiencing tension and three as health strategies. Salutogenic theory seems to be a useful framework for illuminating the health resources and strategies adopted by employed women who become mothers. The identified health resources when experiencing tension and the health strategies applied may have implications for maternity care professionals and employers in promoting the health of such women and supporting them to combine work and family life.

## 1. Introduction

The World Health Organisation (WHO) [1–3] describes the health of women throughout the childbearing years as crucial for the health and development of future generations. The maternal transition that occurs for first time mothers involves a huge amount of personal work [4–6]. However, this transition may differ for women who are employed outside the home, as combining motherhood and work can be challenging [7, 8]. In the last decades, the number of women taking part in paid employment has increased [3, 9, 10]. Employment promotes women's autonomy and health [3, 11, 12] but may equally pose a threat to the health of mothers, due to the difficult demands of balancing motherhood and employment [3, 8, 13–16]. The European Union aims to protect the health of employed pregnant women and supports working mothers to combine work and family life [10]. However, modern working life places significant demands on employees due to greater pressure and responsibility and a high requirement on autonomy and flexibility [17–19]. In Norway, the health of expectant mothers is generally considered good [20]. However, during pregnancy about 60% of employed women are sick-listed [21] and some feel discriminated in terms of work and career [22]. During maternity leave, both a higher risk of depression [23] and an increased life satisfaction are reported [24]. After maternity leave, many reduce their hours of employment [25], which may decrease their satisfaction at work [26, 27] as well as having a negative impact on their family life [28]. Despite the fact that women are encouraged to combine work and family life, it can threaten their well-being when returning to work.

Previous studies of the health of employed women during pregnancy and early motherhood report physical, mental, and social problems [6, 29–32]. Their physical health problems include fatigue and nausea, back pain, pelvic pain, and fear of injury when struggling with awkward physical work tasks, leading to sick leave [29, 31, 32]. Regarding mental and social problems, many pregnant employees report discrimination due to lack of entitlements, insecurity about their future as professionals, challenges due to a great sense of responsibility at work, and difficulties in balancing between the organisation's needs and their own [7, 22]. Preparing for motherhood may involve a huge amount of personal work and changed relationships [6]. One study also indicated that two out of three pregnant employees are in need of job adjustments [33]. Postpartum, employed women are challenged by fatigue, lack of energy [34], being unprepared for the return to work [35], anxiety about not being a good mother [7], and difficulty balancing work and family life [16]. Many devote their time to work and family at the expense of personal care [36], reduce their working hours, change roles and thus experience less opportunities for promotion at work [26, 36]. Nevertheless, social support and relationships at work are described to have a positive influence on balancing work and family life [16, 34]. In summary, most studies indicate that the health of employed women in pregnancy and early motherhood is challenged by health risks. However, some studies [16, 34] describe social support and relationships at work as having a positive influence on health; these could be referred to as health resources (cf. [37]). Other studies [4, 5, 16] explicitly demonstrate that women focus on reestablishing their identity, seek others in similar situations, and reduce their working hours when combining work and family life, all of which could be considered as health strategies (cf. [37]). In order to promote health as well as enhance the balance of work and family life among employed women who become mothers for the first time, more research should place greater focus on health resources and strategies as opposed to health risks. Such understanding is significant for the women, the healthcare system, and employers who are responsible forprotecting employed women's health. The aim of this study was to investigate the health resources and strategies of employed women in Norway during pregnancy and early motherhood by means of salutogenic theory [37, 38].

## 2. Theoretical Background

Antonovsky's salutogenic theory describes ways of maintaining and promoting health and postulates that health is dependent on the individual's generalized resistance resources (GRRs) and sense of coherence (SOC) strategy [37, 38]. In this paper, GRRs are considered an individual's health resources, that is, her/his potential for health and current health status on the continuum of health (cf. [38]). According to Antonovsky [38], the individual's health resources are any individual characteristic that can facilitate tension management. The GRRs are grounded in biological, social, cultural, and historical dispositions, which constitute her/his SOC strategy, namely, how to act to “create health” [37]. The SOC strategy is a mediator of health in life events that involve stress. It implies a global perspective on life, how the individual makes sense of and deals with stressful events, and encompasses three concepts: manageability, comprehensibility, and meaningfulness [37]. The first concept, manageability, refers to load balance, mastery and control, taking part, and making decisions. The second concept, comprehensibility, describes the individual's way of seeing the world as ordered rather than chaotic, viewing any given situation as consistent and predictable as well as her/his participation in shaping the outcome. The third concept, meaningfulness, concerns an individual's view of the importance of participating in shaping outcomes, her/his attitude to responsibility, and the belief that managing tension is worthwhile and meaningful. The SOC strategy is regarded as applicable across cultures. The more evident the SOC components are to an individual, the stronger her/his SOC and the more successful her/his coping and well-being are [37, 38]. However, as health resources and SOC are intertwined, constituting a dynamic, the level of SOC will enhance an individual's health resources, in turn increasing her/his ability to cope. Identifying and supporting an individual's health resources and strategies will be a way to promote her/his well-being.

## 3. Materials and Methods

### 3.1. Design

A hypothetical-deductive hermeneutic interpretive approach inspired by Gadamer was employed [39, 40]. The study is a secondary analysis (cf. [41]) of three previous studies with a qualitative emergent design [42] that explored experiences of employed women who became first-time mothers in Norway [43–45]. In the present study, our assumption was that the women's experiences could be understood in the context of salutogenic theory [37, 38]. We acknowledge that understanding is an act of interpretation and that entering into a dialogue with the material can lead to a higher level of abstraction [40, 46, 47]. Our assumption was that application of salutogenic theory to the previous studies could deepen understanding of how to promote the health of women who become mothers for the first time and how to support them to combine work and motherhood.

### 3.2. Participants and Data Collection in the Original Studies

Data were originally gathered from employed pregnant women (*n* = 10), who were followed during maternity leave (*n* = 9) and after returning to work (*n* = 9). The participants were purposively selected in order to represent the phenomenon under study [42], that is, reproductive health of employed women. The women were invited to take part in the study by midwives at the mother-child health clinic where they had their regular check-ups. The inclusion criteria were women about to become mothers for the first time, being in full time or 75% employment, intending to return to work after maternity leave, low-risk pregnancy, age over 20 years, living with their partner, and being able to speak Norwegian. The data collection in the three initial studies was based on unstructured individual interviews (cf. [48]). The studies were part of a larger qualitative, explorative, and descriptive study of the reproductive health of employed women in Norway [49]. The interviews were audiotaped and transcribed verbatim. The studies were approved by the Norwegian Regional Committee for Research Ethics (number 094.07) and the Norwegian Social Science Data Services (number 16725).

### 3.3. Data Collection

Qualitative data from three datasets on employed women were reanalysed [43–45]. The datasets comprised 28 depersonalised transcripts produced by one of the authors (Marit Alstveit) in 2008 and 2009. The second and third authors were familiar with the original studies (cf. [50]). The first dataset [43] exploring employed pregnant women's experiences of work during pregnancy and expectations of becoming a mother for the first time comprised ten transcripts. The second dataset [44] consisted of nine transcripts of interviews exploring first-time mothers' experiences of social relationships during maternity leave. The third dataset [45] comprised nine transcripts from interviews exploring first-time mothers' return to work.

### 3.4. Data Analysis

The secondary analysis was conducted by utilising data from the three datasets and was supplementary in the sense of investigating aspects that were not central in the original research (cf. [41, 51]). A systematic interpretive process was employed to develop an understanding by means of questioning and answering, that is, a back and forth movement between the parts and the whole (cf. [40, 52]). When reading and interpreting the datasets, the theoretical salutogenic concepts of GRRs and SOC [37, 38] were employed to achieve a hermeneutic horizon. In a deductive way (cf. [53]) while trying to be open-minded, we searched the datasets for material that described the women's health resources and strategies. After interpretation and sorting, meaning units with common elements were formulated into subthemes and then grouped and abstracted into themes (cf. [54]). This was a dialectic process between the datasets from each of the studies, the subthemes, themes, concepts of salutogenic theory, and our preunderstanding [40, 52]. Finally a synthesis emerged which is reported in the present study.

### 3.5. Trustworthiness

The process of refining and validating the findings involved collaboration between all the researchers (cf. [42]). The synthesised understanding implied a fusion of horizons in relation to the researchers' preunderstanding, theoretical perspectives, and previous research. The reanalysis was conducted by the researchers responsible for the original studies (Marit Alstveit, Elisabeth Severinsson, and Bjørg Karlsen). The interpretation was influenced by the context, the researchers' preunderstanding, that is, as nurses, mothers, and familiarity with Scandinavian culture (cf. [42]). Descriptions of the cultural context in which the data were generated, characteristics of the sample, the analytic process, and how the interpretations were arrived at, including quotations that enable direct access to the data, are provided to enhance the trustworthiness of the findings [55, 56].

## 4. Results 

The participants in the original studies were women living with a partner in Norway, aged between 21 and 35 years (mean = 29.7 years), in full-time employment and experiencing a low risk pregnancy at the time of recruitment to the study. They had been employed for 1.5 to 8 years (mean = 4.5) and worked in different public and private organisations, as well as in large and small companies. Five had jobs that involved physical work; one was a manager and all intended to return to work after maternity leave. All but one had a third level education.

**Figure 1:**
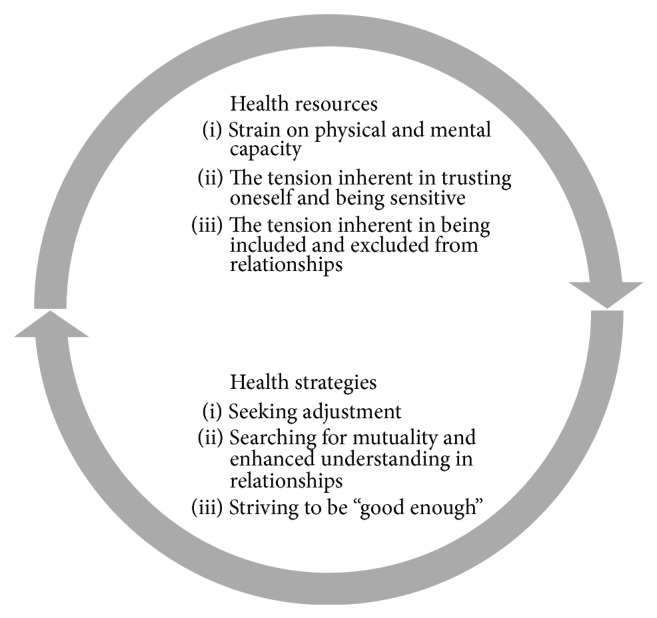
Overview of health resources and strategies during pregnancy and early motherhood.

In total, six themes were identified in the datasets from the original studies; three were interpreted as health resources when experiencing tension and three as health strategies used by the women. The health resources were grouped according to characteristics of the women's GRRs [38] and comprised “strain on physical and mental capacity,” “the tension inherent in trusting oneself and being sensitive,” and “the tension inherent in being included in and excluded from relationships.” Health strategies were grouped in accordance with the SOC components [37] and condensed into the following themes: “seeking adjustment,” “searching for mutuality and enhanced understanding in relationships,” and “striving to be ‘good enough.'” An overview of the interpreted health resources and strategies is presented in Figure 1.

### 4.1. Health Resources

#### 4.1.1. Strain on Physical and Mental Capacity

The first theme identified in the datasets was “strain on physical and mental capacity.” In pregnancy, the participants described “living on the edge of being overstretched,” “being exhausted by adapting to professional life,” and “believing motherhood to involve a great deal of responsibility.” Most women reported having a body and mind that did not cope as before and were more vulnerable to stress, as they had to organise and finish their work before the start of their maternity leave. One woman expressed: “*I had to accept the fact that I was more worn out than I thought … but believed that I had to go on. There were so many duties to finish and delegate.*” In early motherhood, the women seemed to be in an unknown and limited world, lost, and unsure about how to best take care of their child. When on maternity leave, several participants reported feeling ineffective and in a state of stagnation. Some described that their career had stopped in relation to that of colleagues and partners. During pregnancy and early motherhood several women expressed feeling less flexible due to being responsible for their work and the well-being of the foetus/child. One woman reported: “*The situation is so vulnerable, he (the child) has been sick, I have been sick … it has been stressful–a heavy workload, and I no longer have the resources to manage.*” It seemed difficult for the women to take care of their own needs, those of the child, the relationship with their partner, and the workplace.

#### 4.1.2. The Tension Inherent in Trusting Oneself and Being Sensitive

The second theme identified was “the tension inherent in trusting oneself and being sensitive.” During pregnancy, most of the participants reported trusting their ability to manage the unknown. Some described having no choice other than to manage as both a mother and an employee. As mothers, several stated that they received a great deal of advice but had to trust their own judgement about which to follow. It seemed as if trust in and knowledge of oneself developed. One woman reported: “*You learn to know yourself better, about your capacity and the weaknesses you thought you had, but did not have after all.*” The women described a balance between sensitivity and self-confidence. In pregnancy, one participant expressed: “*It's like becoming more sensitive […] when being unable to take part…. I can feel a bit like a ball and chain.*” The women also seemed sensitive about how others responded to them, their child, and their care of the child. On their return to work after maternity leave, several appeared to wonder how they were perceived at the workplace but stated that they could express their needs to a greater extent than before.

#### 4.1.3. The Tension Inherent in Being Included in and Excluded from Relationships

The third theme that emerged was “the tension inherent in being included in and excluded from relationships.” During pregnancy, several of the participants described that their condition was ignored by colleagues and their employer. When planning for maternity leave, many expressed that they wanted to have a life outside the family. With regard to social relationships at the workplace, one woman commented: “*I don't want to vanish and be forgotten by the team and then suddenly come back.*” On maternity leave, several stated that they were unsure about what kind of contact the workplace expected. Visiting the workplace gave rise to a feeling of causing an interruption as well as being valued as more than a mother. Being invited to social gatherings at work was reported to create a sense of belonging and being part of the workplace. As mothers, all the participants stated that they missed the company of other adults and several felt lonely. One participant commented: “*It's nice to be together with the child, but I need something else as well … it does not take long before you feel that you are isolated. I miss meeting other people, you need more than just your family.*” In addition, the relationship with their partner seemed to suffer. The women described how their partner acted as a father, considered their needs, did not place full responsibility for the child on them and how they as a couple managed being parents. These experiences seemed related to the feeling of being a “team.” One woman stated: “*I feel we have become closer to each other, like a family, we have become ‘us.'*” Meeting other mothers was described as confirming them as mothers and preventing them from feeling lonely.

### 4.2. Health Strategies

#### 4.2.1. Seeking Adjustment

During pregnancy most of the participants strived to adapt, managing duties at work despite a body and mind that did not cope as before, as well as finish their work and hand over to colleagues before maternity leave. One woman stated: “*At the end it was only work and then home on the bus. There was no time for anything else. I needed to discipline myself and scale down, I had to restrict myself more.*” It seemed that signals such as fatigue from their body or movement of the foetus when stressed made them seek adjustment. In early motherhood, they gradually learned how to care for the child, adjusting to the rhythm of caring, being occupied and having little time for themselves. On their return to work, most of the participants strived to manage the workload and readjust their lives in the tension inherent in work and motherhood. Having enough time together with the child seemed to be central. Several participants changed workplace to save time when taking the child to day care, reduce the time spent commuting, and avoid overtime. The new workplace was chosen primarily because of the ability to combine the workload with the care of the child, not for career reasons. In addition, several women reported changing their ways of performing duties at work, reducing the amount of social contact with colleagues as well as increasing the planning and structure of their work in order to become more effective. They seemed less flexible in terms of devoting time to work but more flexible in performing their duties. However, if the women had an opportunity to bring work home they took it. One participant commented: “*I'm not in the office as much as I used to be … I do the required number of hours. I prefer to work after I've put him (the child) to bed in the evening.*”

#### 4.2.2. Searching for Mutuality and Enhanced Understanding in Relationships

The fifth theme identified in the transcripts was “searching for mutuality and enhanced understanding in relationships.” Several women reported reading the literature about pregnancy and motherhood. However, most expressed that the experiences of friends and family members were essential for understanding motherhood and childcare. Furthermore, their own experiences of not always being in control during pregnancy and motherhood seemed to increase their understanding of colleagues with health problems. A woman stated: “*I feel that I can understand the situation of others better, in a strange way … something has happened.*” However, several women described lack of understanding from their employer and colleagues concerning their need for adaptation at work. Meanwhile, when on maternity leave, most reported that sharing the company and experiences of other mothers was of vital importance. Meeting other mothers with whom they could identify and who understood their experiences seemed to confirm them as mothers. One woman expressed: “*Meeting in postnatal groups is very useful. I needed to see that there are different ways of caring and no recipe … it is important to listen to other mothers in the same situation.*” However, in relation to their partner, the women reported both a sense of mutuality and a differing understanding of how to be an employed mother and a responsible parent. Some couples seemed to disagree about how to plan their time, as reported by one participant: “*I work in the evening and he babysits, but my free time is gone and he uses his for football matches.*” Having someone with whom to share experiences appeared important for the women's understanding.

#### 4.2.3. Striving to Be “Good Enough” 

The last interpreted theme was “striving to be ‘good enough,'” which concerned how the participants evaluated their situation. During pregnancy, some women reported feeling degraded or ignored when being unable to perform their work as before or when their needs as an expectant mother were not acknowledged. One woman expressed: “*When I delivered my sick leave ‘cert', they did not ask me what was wrong, so I felt a bit ignored, as if I was not very important.*” However, during pregnancy most women expressed that when they became a mother they would be valuable in another way. After becoming a mother, several experienced a new dimension in life. Nevertheless, many felt they had failed if they were unsuccessful at breastfeeding or comforting the child. On the other hand, a satisfied and smiling child gave them a feeling of success. On returning to work, several participants seemed to struggle with the sense of not being a good enough mother and failing to take responsibility for the child's best interest. They worried about the day care and argued that the kindergarten did not provide the same care as they did. On the other hand, they regarded the Kindergarten as favourable for the child's development. It seemed that several were striving to combine being a good mother and a good employee. One woman who had flexible working hours expressed: “*When I'm at home with the child I feel guilty about not being at work and when I'm at work I feel guilty about not being at home with the child.*” Some revealed that they no longer devoted themselves to the workplace as much as previously. Not being as accessible as before made them worry about how they were perceived as employees. One participant commented: “*You do not want to be a burden because you have a child, someone who is unreliable […] you should be accessible both physically and mentally … I have experienced that due to sick leave I needed to make clear that this is not how I used to be.*”

## 5. Discussion

In this study, we interpreted and identified the health resources and strategies of employed women during pregnancy and early motherhood by means of salutogenic theory.

### 5.1. Main Results

The findings indicate that the health resources of employed women appear to be challenged in the sense of “strain on physical and mental capacity,” “the tension inherent in trusting oneself and being sensitive,” and “the tension inherent in being included in and excluded from relationships.” The women described tension in the areas of physical, mental, ego-identity, and interpersonal relationships, reflecting how they experienced adverse events. Their health seemed challenged by more than pregnancy and motherhood, involving self-understanding and sensitivity pertaining to fulfilling the expectations of others. These findings are in accordance with several studies by international researchers [16, 29, 34] describing the risk to health in these areas. On the other hand, our findings may reflect that although the women are challenged by living with tension, they have health resources to manage this tension in order to improve health. This is consistent with the salutogenic approach suggesting that health resources at tension might create opportunities as well, if dealt with successfully. According to Antonovsky [37, 38], health resources may foster life experiences and contribute to a strong SOC, if such experiences are predictable and not too frustrating. In turn, a strong SOC enables an individual to successfully deal with adverse life events and can promote health resources. Moreover, our findings revealed health strategies such as “seeking adjustment,” “searching for mutuality and enhanced understanding in relationships,” and “striving to be ‘good enough.'” The first theme “seeking adjustment” reflects the manageable component of SOC. Many of the participants in our study described this strategy as essential for planning, making decisions, and becoming more effective as a mother and an employee. These findings may reflect the use of active coping strategies to balance work and family life. However, our findings are inconsistent with the results of other studies, in which strategies such as sick leave and reduction of working hours [36, 57] are described as coping strategies, which could indicate that employed women have abandoned their professional ambitions. The second health strategy “searching for mutuality and enhanced understanding in relationships” refers to the SOC concept of comprehensibility and reflects the importance of supportive interpersonal relationships for success at work and in private life. This finding accords with previous research describing the importance of social support for well-being and for balancing work and family life [16, 58], reflecting that such support could help the women to view their situation more predictably and consistently. The third strategy “striving to be ‘good enough'” reflects the meaningful component of SOC and deals with how the participants evaluated their situation. Some of the women described a conflict between wishes and capacity, which could reflect their feeling of not being good enough. This is consistent with findings from two previous studies indicating that employed pregnant women and mothers experience such a conflict (cf. [59, 60]). If the women's strategies are not successful, this can have a negative impact on their health resources as well as their motivation as employees (cf. [61, 62]). Moreover, the findings of tension related to trusting oneself, being sensitive, and striving to be “good enough” may indicate development of the women's moral identity and competence [63], reflecting health resources and strategies that are enriched when combining motherhood and work (cf. [64]).

It should not be left to each woman to figure out how to take care of her well-being and balance work and family life at this vulnerable period. By applying the salutogenic approach and identifying the women's health resources and strategies, maternity care professionals could have a positive influence on employed women's well-being and ways of balancing work and family life [65, 66]. This could include assisting them in recognising and becoming aware of their competence [59, 60, 63], supporting their strategies and guiding them to achieve practical adjustments. The employer could be made aware of the tension to which the women may be subject that jeopardizes health as well as strategies that can promote conditions favourable to health [19]. Maternity care professionals could contribute to a discourse about an inclusive working life (cf. [67]) that might assist women in pregnancy and early motherhood. The demands of modern working life [62, 68, 69] and the cultural expectations on employees and mothers may not be compatible [15, 70]. Maternity care professionals should therefore consider ways of enhancing public awareness by informing society that pregnancy and early motherhood are “another” state of normality and that women should not be expected to be “ultrafit” employees at this time (cf. [59]).

### 5.2. Strength and Limitations

Secondary analysis is a useful method in health research, as it ensures that data are used effectively [41] and reduces the burden on participants [71]. The data utilised in this study were originally generated by in-depth interviews to explore experiences of employed women who became mothers for the first time. Questions about health resources and strategies were not specifically focused on in the original data collection. The reason for posing questions about health resources and strategies and applying Antonovsky's salutogenic theory of interpretation was to achieve a more profound understanding of the women's situation [37, 38, 72]. Reusing qualitative data and interpreting the three independent datasets by means of the salutogenic theory were found to add significant knowledge of the health resources and strategies of employed women who became mothers for the first time. This might indicate that the data sets and the theoretical frame were relevant to the research question [46]. However, data are not reports of preexisting reality but are “coconstructed” to fit the purpose of the project in which they are used. Sandelowski [46] argued that reusing data does not differ from employing them in the primary analysis because data are situated and new interpretation of them can always be made. When analysing the data, the strength was that the researchers were familiar with the context in which the data were generated, as they were responsible for the data collection in the original studies [41]. Sandelowski [46] warned against using secondary analysis in a way that could harm the participants, especially when the content of the analysis is far removed from what the participants consented to. This study was a continuation of the original analyses, but with a new framework. The same researchers handled the data and the analysis did not differ from what the participants had consented to in relation to the previous studies.

## 6. Conclusion

Salutogenic theory seems to be a useful framework for illuminating the health resources and strategies adopted byemployed women who become mothers. The identified health resources when experiencing tension and the health strategies applied to manage this tension may have implications for maternity care professionals and employers in promoting the health of such women and supporting them to combine work and family life.
